# Epigastric pain of unknown origin, esophageal and gastric involvement: a Henoch-Schönlein Purpura case report

**DOI:** 10.3389/fmed.2025.1577291

**Published:** 2025-04-16

**Authors:** Mengmeng Li, Chaoyuan Huang, Hailun Xing, Shijuan Luo, Yiqun Lin, Yuxiang Kuang, Jing Wang, Suiping Huang, Zhenhao Ye

**Affiliations:** ^1^The Second Clinical College of Guangzhou University of Chinese Medicine, Guangzhou, Guangdong, China; ^2^Department of Gastroenterology, The Second Affiliated Hospital of Guangzhou University of Chinese Medicine, Guangzhou, Guangdong, China; ^3^Department of Gastroenterology, Guangdong Provincial Hospital of Chinese Medicine, Guangzhou, Guangdong, China

**Keywords:** Henoch-Schönlein Purpura, intramural hematoma of the esophagus, gastric mucosal erythema, abdominal pain, epigastric pain

## Abstract

Henoch-Schönlein Purpura (HSP) is an acute systemic vasculitis that primarily affects the skin, gastrointestinal tract, kidneys and joints. It predominantly occurs in children but can occur at any age. We report a case of 48-year-old male patient suffering from unexplained epigastric pain alternating between relapses and remissions. The patient’s initial clinical manifestation was atypical, starting with severe epigastric pain, without cutaneous purpura, and with normal rheumatologic and immunologic markers, making diagnosis difficult. The patient was eventually diagnosed with HSP. This case highlights the importance of considering this diagnosis in patients with unexplained abdominal pain and emphasize the role of endoscopy in diagnosing complex cases.

## 1 Introduction

HSP is a systemic small-vessel vasculitis that mainly involves small blood vessels, typically characterized by non-thrombocytopenic palpable purpura, with cutaneous purpura, abdominal pain, arthralgia/arthritis, hematuria and proteinuria as the main clinical manifestations (1–3). The disease is most commonly seen in children and adolescents and is relatively rare in adults. The annual incidence rate in adults is 0.8–1.8 per 100,000 (4). The etiology and pathogenesis remain incompletely understood but may be related to bacterial or viral infections, parasitic infections, food allergy, drugs, diabetes mellitus, immune complex deposition, and complement activation (5, 6). Since there are no specific diagnostic markers for HSP, it is difficult to make a correct diagnosis based on the symptoms, signs, laboratory and imaging tests prior to the onset of skin purpura in patients (7).

Gastrointestinal-predominant Henoch-Schönlein Purpura, which mainly involves the gastrointestinal tract, skin and other organs, often begins with gastrointestinal symptoms including abdominal pain, vomiting and hematochezia (8). If cutaneous purpura is the first symptom, it is easy to confirm the diagnosis. However, for those who have gastrointestinal symptoms such as abdominal pain, vomiting and hematochezia as the first manifestation are easily misdiagnosed with acute gastroenteritis, peptic ulcer, gastrointestinal bleeding and other diseases.

Our patient’s initial presentation was characterized by severe epigastric pain, which recurred over the last 5 months and was not diagnosed until the appearance of purpuric rash. The gastrointestinal symptoms preceded the skin purpura, making the diagnosis of HSP more difficult. However, gastroscopy had already revealed signs of HSP. Diagnosis delay leads to effective treatment delay. Therefore, timely diagnosis and appropriate treatment are essential to alleviate the patient’s suffering.

## 2 Case report

**Figure 1:**
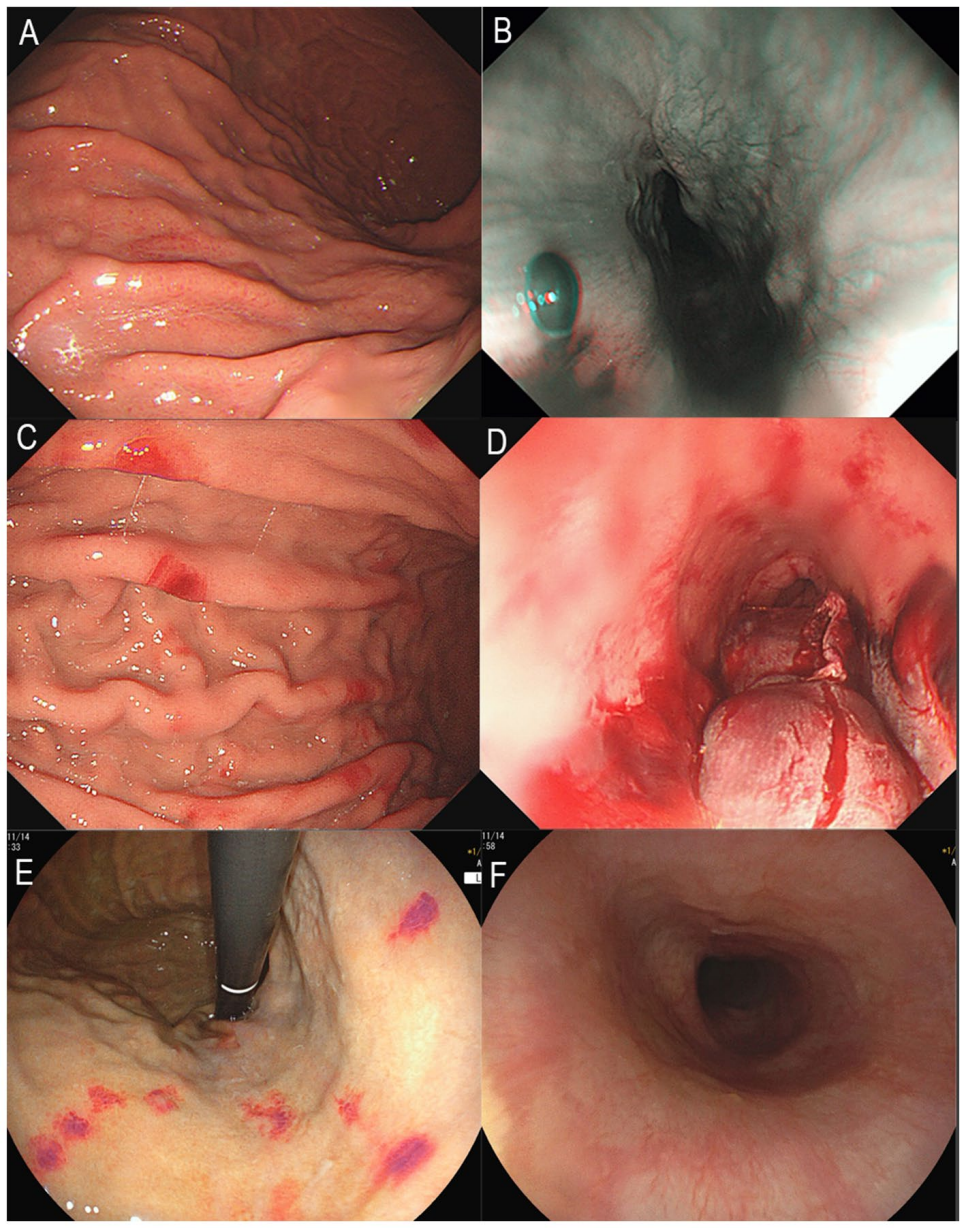
Upper gastrointestinal endoscopy images. **(A)** Gastric mucosa with spotty redness, August 2024. **(B)** Submucosal hematoma in the lower esophagus, August 2024. **(C)** Scattered erythema on the gastric mucosa, September 2024. **(D)** Rupture and bleeding of hematoma in the lower esophagus, September 2024. **(E)** Multiple erythema on the gastric mucosa, November 2024. **(F)** Normal mucosa in the lower esophagus, November 2024.

A 48-year-old male was admitted for the first time due to relapsing and remitting severe epigastric pain over the past several months on August 19, 2024. The pain was accompanied by vomiting, acid reflux and heartburn, but was unrelated to meals. Medical history included hypertension, diabetes, gallstones and diaphragmatic hernia. Medications consisted of bisoprolol fumarate, felodipine and insulin. During hospitalization, he denied fever, recent dietary or medication changes, heavy metal exposure history and skin purpura. Given his symptoms, we promptly conducted relevant examinations (Table 1). Fecal occult blood test was positive (2+); D-dimer levels fluctuated between 0.78 and 1.42 mg/L FEU (normal range 0.00–0.50 mg/L FEU). The white blood cell count fluctuated between 5.45 and 15.48 × 10^9^/L (normal range 3.50–9.50 × 10^9^/L). Hemoglobin and platelet count, urinalysis, liver function, renal function, myocardial enzyme profile, hypersensitive troponin T, lipids, amylase, lipase, C-reactive protein (CRP), erythrocyte sedimentation rate (ESR), procalcitonin and food allergen IgG were unremarkable. Bacterial, parasitic, tuberculosis tests and tumor markers were negative. Rheumatologic and immunologic markers were all within the normal reference range. Electrocardiogram, chest X-ray, computed tomography of chest and abdomen were unremarkable. Upper gastrointestinal endoscopy showed chronic gastritis (Figure 1A), and gastric biopsies revealed chronic mucosal inflammation. Colonoscopy showed no abnormalities. On admission, he received phloroglucinol, ketorolac tromethamine, tramadol, pethidine for pain relief, but his symptoms did not alleviate significantly. The patient also received a treatment of 40 mg of methylprednisolone for 3 days, with suboptimal results. Additionally, we organized two multidisciplinary consultations involving the following departments: Gastroenterology, Gastrointestinal Surgery, Hepatobiliary Surgery, Endocrinology, Cardiovascular Medicine, Radiology, Anesthesiology, Psychology, Neurology, and Rheumatology, but the cause of the patient’s epigastric pain remained unidentified at discharge. We still lean toward an immune-related disease.

**Table 1:** Summary of patient’s laboratory test results.

Variables	August 2024	September 2024	November 2024	Reference range
White blood cell count (×10^9^/L)	5.45–15.48	5.20–10.18	8.97–18.99	3.50–9.50
Hemoglobin (g/L)	130–142	117–142	137–140	130–175
Platelet (×10^9^/L)	279–308	211–253	212–225	125–350
Fecal occult blood test	2+			Negative (−)
Urine protein (/HP)	Negative (−)	1+	1 + −2+	Negative (−)
Urine microalbumin (mg/L)		129.00		0–30
ALT (U/L)	19–26	31–34	80	9–50
AST (U/L)	15–17	15–18	33	15–40
Creatinine (μmol/L)	63–86	84	59–69	57–97
D-dimer (mg/L FEU)	0.78–1.42	0.94–1.99	0.47–0.50	0.00–0.50
Blood lactate (mmol/L)	2.00–6.11	1.67–4.28		0.50–2.20
Lipase (U/L)	31	27–141	68	13–60
Amylase (U/L)	89	12–67	20	35–135
Erythrocyte sedimentation rate (mm/h)	2–8	5		0–20
Procalcitonin (ng/mL)	0.05	0.03	<0.02	0.00–0.50
C-Reactive protein (mg/L)	0.80–1.18	0.63		0.00–6.00
Hypersensitive troponin T (μg/L)		0.018	0.010–0.011	0.00–0.014
Serum amyloid A (mg/L)	<10.00		11.43	<10.00
Total immunoglobulin E (IU/mL)	21.83	20.83	21.11	0–100
Rheumatologic and immunologic markers				
Immunoglobulin A (g/L)	1.61	1.91	1.64	1.00–4.20
Immunoglobulin G (g/L)	8.50	10.60	10.10	8.60–17.40
Immunoglobulin M (g/L)	0.39	0.61	0.63	0.30–2.20
Complement C3 (g/L)	0.89	0.74	0.79	0.7–1.4
Complement C4 (g/L)	0.30	0.24	0.23	0.1–0.4
Anti-streptolysin O (IU/mL)		50		0–200
Rheumatoid factor (IU/mL)		<10		0–14
Antinuclear antibody	Negative (−)			Negative (−)
Quantitative anti-PR3 ANCA	<2.30	<2.30	<2.30	0.00–20.00
Tumor markers				
CA19-9 (U/mL)	10.70			0.00–30.00
CEA (ng/L)	1.42			0.00–5.00
AFP (ng/L)	3.49			0.00–7.00
Pathogen measurement				
Tuberculosis antibody	Negative (−)			Negative (−)
Interferon-gamma release assay (IGRA) for tuberculosis	Negative (−)			Negative (−)
Lung parasite antibody	Negative (−)			Negative (−)
Liver parasite antibody	Negative (−)			Negative (−)
Brain parasite antibody	Negative (−)			Negative (−)
EB-DNA			Negative (<5.0E+02)	Negative (<5.0E+02)
CMV-DNA			Negative (<5.0E+02)	Negative (<5.0E+02)
Weil-Felix reaction			<1:40	0–1:160
Widal reaction			<1:40	H: 0–1:160 O, A, B, C: 0–1:80

**Figure 2:**
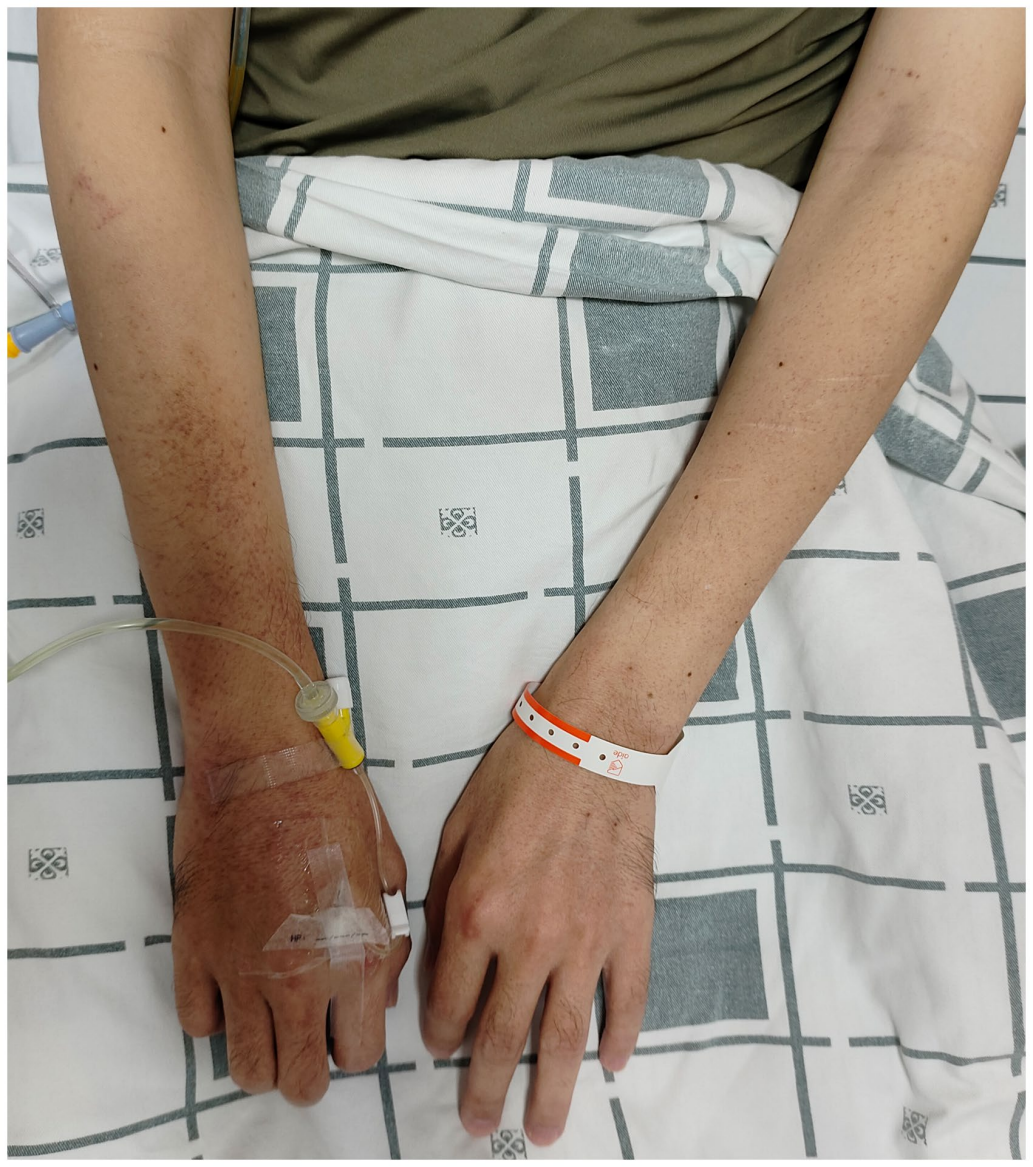
Image of purpura on the upper limbs skin.

The patient was readmitted on September 16, 2024, due to severe epigastric pain and scattered dark purple hemorrhagic spots visible on both upper limbs (Figure 2). Laboratory tests (Table 1) revealed a white blood cell count of 10.18 × 109/L (normal range 3.50–9.50 × 109/L), D-dimer of 1.99 mg/L FEU (normal range 0.00–0.50 mg/L FEU), blood lactate of 4.28 mmol/L, urine protein of 1+, urine microalbumin of 129 mg/L (normal range 0-30 mg/L). CRP, ESR, amylase, liver tests, creatinine, electrolytes were unremarkable. Given the presence of purpura on upper limbs of the patient, we invited a dermatologist for consultation to assist in confirming the diagnosis. Based on the American College of Rheumatology criteria for HSP (1990) (9): (1) age<20 years at disease onset; (2) palpable purpura; (3) acute abdominal pain; (4) biopsy showing granulocytes in the walls of small arterioles or venules, the patient’s history of upper limbs purpuric rash, recurrent epigastric pain, and fecal occult blood test positive suggested a diagnosis of HSP. He was treated with methylprednisolone sodium succinate (120 mg), anticoagulation with nadroparin calcium, and gastroprotective and analgesic therapies. However, methylprednisolone sodium succinate and nadroparin calcium had to be discontinued due to the presence of bright red drainage fluid in the gastric tube. Emergency gastroscopy was performed, indicating bleeding from intramural hematoma of the esophagus (IHE) (Figure 1D), and gastroscopic hemostasis was performed, and the gastric mucosa showed scattered erythema and speckle congestion (Figure 1C), which was consistent with the endoscopic manifestations of HSP. Ultimately, epigastric pain due to HSP was diagnosed. His symptoms were notably reduced on the first postoperative day, completely relieved on the second day. Upon reviewing the initial gastroscopy, we discovered an IHE (Figure 1B), which we had overlooked.

The patient was readmitted for the third time on November 6, 2024, due to severe epigastric pain preceded by purpura on both upper limbs. Gastroscopy revealed the patient’s IHE was absorbed (Figure 1F), multiple punctate and patchy purpuric lesions in the stomach (Figure 1E), and pathology confirmed chronic inflammation of the mucosa. Colonoscopy suggested colonic polyps, which were removed and confirmed as hyperplastic polyps. No significant abnormalities were observed in Double-balloon enteroscopy. The patient’s epigastric pain was alleviated after a 7-day course of intravenous methylprednisolone (120 mg daily). After discharge, the patient was switched to oral methylprednisolone and the dose was gradually reduced until it was completely discontinued. By the 12-week follow-up, the patient had no episodes of epigastric pain. He was suggested to have a regular visit and seek a doctor immediately if melena or epigastric pain occurs.

## 3 Discussion

HSP is an acute systemic vasculitis that primarily affects the skin, gastrointestinal tract, joints, and kidneys (10, 11). The characteristic skin manifestation is non-thrombocytopenic palpable purpuric rash, which most commonly affects the lower limbs and buttocks but can also involve the trunk and upper limbs (12). Notably, the gastrointestinal symptoms may precede the skin lesions (13). Gastrointestinal involvement is common, presenting with abdominal pain, vomiting and gastrointestinal bleeding (8, 14). Joint symptoms are non-erosive arthralgia and swelling, predominantly affecting the knees and ankles (8). Renal involvement is more common and severe in adults, manifesting as hematuria and proteinuria (15). In addition, orchitis, cerebral vasculitis, interstitial pneumonia or pulmonary fibrosis have been reported (16–18). HSP is difficult to diagnose based on symptoms, laboratory tests, and examination results before the onset of purpura.

This case is atypical. First, gastrointestinal symptoms preceded typical purpuric rash and gastroscopic features (IHE and gastric mucosal erythema) by 5 months. Second, typical cutaneous purpura usually occurs on the buttocks and lower limbs, but in our patient, the purpura appeared on upper limbs.

For patients with unexplained abdominal pain, the possibility of HSP should consider during diagnosis, and endoscopy should be considered. Endoscopy is highly effective for diagnosing HSP when it involves the gastrointestinal tract (19). Mucosal lesions in patients with HSP can occur at any site within the gastrointestinal tract. The lesions visible under endoscopy include erythema, edema, petechiae, erosions, ulcerations, nodular changes, hematoma-like protuberances, and stenosis (20, 21). A study of gastroscopy in 48 patients with HSP showed that the second part of the duodenum is the most frequently affected site in the upper gastrointestinal tract by HSP, with an incidence rate of 52.9% (20). The other sites in descending order of frequency are the duodenal bulb (41.3%), gastric body (25.4%), gastric antrum (23.1%), gastric fundus (3.8%), and esophagus (3.3%). A study of colonoscopy in 19 patients with HSP showed that the rectum (80%) and terminal ileum (60.1%) were the most commonly affected sites, followed by the cecum (5.1%), ascending colon (4.5%), descending colon (4.5%), and transverse colon (3.7%) (20). Esaki et al. reviewed the gastrointestinal endoscopy results of seven patients with HSP and found that the duodenum and small intestine were the most frequently affected sites, with none of the patients showing involvement of the esophagus (22). Esophageal involvement is rare, but it can occur in the course of HSP. Deguchi et al. presented two cases of HSP involving the esophagus, with upper endoscopy showing linear ulcers in the mid esophagus in both cases (23).

Upon reviewing this case, we observed that the patient’s gastroscopy consistently revealed gastrointestinal vascular exudative changes during each episode, including IHE and gastric mucosal erythema. The first hospitalization gastroscopy at our hospital showed IHE. The second hospitalization gastroscopy indicated rupture and bleeding of the IHE, with multiple erythematous changes in the stomach. The third hospitalization gastroscopy showed multiple punctate and patchy erythematous changes in the stomach. These results suggest the possibility of HSP in patients to some extent. This case suggests that when endoscopic examination reveals lesions such as hematoma and erythema in the gastrointestinal mucosa, the possibility of HSP diagnosis should be considered, even if cutaneous purpura is not yet present.

## 4 Conclusion

We report a rare case of HSP of epigastric pain of unknown origin, esophageal and gastric involvement. This case indicates that patients with HSP may experience changes such as IHE and multiple erythematous lesions of the gastric mucosa. Our aims are to raise awareness among physicians about HSP and to emphasize the value of gastrointestinal endoscopy in complex cases which cutaneous purpura is not apparent.

## Data Availability

The original contributions presented in the study are included in the article/supplementary material, further inquiries can be directed to the corresponding authors.
